# Lung-protective ventilation suppresses plasma levels of cell-free DNA in porcine experimental postoperative sepsis

**DOI:** 10.1186/cc13482

**Published:** 2014-03-17

**Authors:** A Nyberg, J Sperber, M Lipcsey, J Jylhävä, M hurme, M Castegren

**Affiliations:** 1 Centre for Clinical Research Sormland, Uppsala University, Eskilstuna, Sweden; 2 Uppsala University, Uppsala, Sweden; 3 University of Tampere, Finland

## Introduction

Mechanical ventilation affects systemic inflammation where protective ventilation attenuates the response [1]. Plasma levels of cell-free DNA (cfDNA) increase and have prognostic value in sepsis [2]. In the present study, the effect of tidal volume and PEEP on arterial and transorgan levels of cfDNA was investigated in a porcine postoperative sepsis model.

## Methods

Two groups of anaesthetised pigs were ventilated with either protective ventilation (VT 6 ml/kg, PEEP 10 cmH_2_O; *n *= 20) or controls (VT 10 ml/kg, PEEP 5 cmH_2_O; *n *= 10) for 7 hours. An artery, the hepatic vein, the portal vein and the jugular bulb were catheterized. Continuous endotoxin infusion at 0.25 μg/kg/hour for 5 hours was started after 2 hours of laparotomy that simulated a surgical procedure.

## Results

The group receiving protective ventilation showed lower levels of cfDNA in arterial blood compared with controls (*P *= 0.02). Trans- hepatic levels of cfDNA were higher compared with trans-splanchnic levels during the experiment (*P *= 0.02), but this effect was attenuated in the group receiving protective ventilation. No difference between the groups was detected in blood samples from the jugular bulb.

## Conclusion

In experimental postoperative sepsis, protective ventilation suppresses arterial levels of cfDNA. The liver seems to be a significant contributor to systemic cfDNA levels, an effect that is suppressed during protective ventilation.
